# Mosaic Loss of Chromosome Y in Peripheral Blood Cells from Whole‐Genome Sequencing and Alzheimer’s Disease in the Amish

**DOI:** 10.1002/alz.089681

**Published:** 2025-01-03

**Authors:** Yeunjoo E. Song, Renee A. Laux, Sarada L. Fuzzell, Sherri D. Hochstetler, Kristy L. Miskimen, Audrey Lynn, Weihuan Wang, Leighanne R. Main, Ping Wang, Yining Liu, Noel C. Moore, Michael B. Prough, Daniel A. Dorfsman, Laura J. Caywood, Jason E. Clouse, Sharlene D. Herington, Alex V. Gulyayev, Susan H. Slifer, Larry D. Adams, Jeffery M. Vance, Michael L. Cuccaro, Paula K. Ogrocki, Alan J. Lerner, Margaret A Pericak‐Vance, William K. Scott, William S. Bush, Jonathan L. Haines

**Affiliations:** ^1^ Department of Population and Quantitative Health Sciences, Case Western Reserve University School of Medicine, Cleveland, OH USA; ^2^ Cleveland Institute for Computational Biology, Case Western Reserve University, Cleveland, OH USA; ^3^ Department of Population and Quantitative Health Sciences, Case Western Reserve University, Cleveland, OH USA; ^4^ Department of Genetics and Genome Sciences, Case Western Reserve University School of Medicine, Cleveland, OH USA; ^5^ Department of Nutrition, Case Western Reserve University School of Medicine, Cleveland, OH USA; ^6^ John P. Hussman Institute for Human Genomics, University of Miami Miller School of Medicine, Miami, FL USA; ^7^ Dr. John T. Macdonald Foundation Department of Human Genetics, University of Miami Miller School of Medicine, Miami, FL USA; ^8^ Department of Neurology, Case Western Reserve University School of Medicine, Cleveland, OH USA; ^9^ Case Western Reserve University School of Medicine, Cleveland, OH USA; ^10^ 1501 NW 10th Avenue, Miami, FL USA; ^11^ John P. Hussman Institute for Human Genomics, Miller School of Medicine, Miami, FL USA

## Abstract

**Background:**

Mosaic loss of chromosome Y (mLOY) refers to acquired aneuploidy in a fraction of somatic cells. In aging men, this has been suggested as a possible biomarker for increased risk of numerous diseases, including Alzheimer’s disease (AD). We investigated mLOY estimated from whole genome sequencing (WGS) as a risk factor for AD in the Midwestern Amish, a founder population with homogeneous lifestyle, reducing the effect of confounding environmental factors.

**Method:**

The calling of mLOY was performed using the Mosaic Chromosomal Alterations (MoChA) pipeline, utilizing long‐range phase information to search for imbalances between maternal and paternal allelic fraction in a cell population. A loss was called when the slope of coverage over allelic deviation was ‐0.94. The cognitive status of each individual was assigned via consensus review of medical history and neuropsychological testing. The cognitively‐impaired (CI) group combined AD, MCI and Unclear individuals and compared to the cognitively‐unimpaired (CU) group. Individuals in 3 age groups (65‐74, 75‐84, ≥85) were included. To evaluate the association between mLOY and risk of developing CI in follow‐up, we fit a Cox proportional hazards model associating mLOY to years of disease‐free follow‐up within individuals for whom blood was sampled prior to CI diagnosis.

**Result:**

After extensive QC, 457 males (mean age = 80.48±5.12) were included. mLOY frequency increased with age (p = 0.029): 18.75% (65‐74; n = 48), 31.86% (75‐84; n = 317) and 39.13% (85+; n = 92). A subset of 372 males were assigned to the CU (n = 202) or CI (n = 170) group and mLOY was slightly higher in the CI group (26.2% in CU vs. 34.1% in CI; p = 0.099). Within 217 males for whom blood was sampled *a priori*, there were 48 subsequent CI events. mLOY was associated with an increased risk of CI diagnosis with hazard ratio (HR) = 1.90 (1.03‐3.56, p = 0.04). After adjusting for the age of sampling, HR increased to 2.31 (1.23‐4.36, p = 0.009).

**Conclusion:**

We assessed the impact of mLOY in males on the risks of CI. We observed the same trend as reported in other European descent populations: mLOY increased with age and the higher sensitivity with WGS data compared with array‐based data. We found that carriers of mLOY had an increased risk of impairment.

